# Supplementary material for: Reproducibility and Relative Validity of a Short Food Frequency Questionnaire for Chinese Older Adults in Hong Kong
Source: Nutrients. 2024 Apr 11;16(8):1132. doi: 10.3390/nu16081132 (PMC11054710; doi:10.3390/nu16081132)
Supplement: Supplementary file 1 [file nutrients-16-01132-s001.zip › Table S1.pdf]

Table S1. The agreement between FFQ1 and three-day dietary records by Bland–Altman analysis.

Parameters	Crude		
	Mean	95% LOA	Rate (%)*
Total dietary fiber (g)	1.17	-6.13, 8.48	5.05
Total sugar (g)	-0.48	-30.41, 29.44	3.54
Saturated fat (g)	2.26	-6.47, 10.99	5.56
Trans fat (g)	-0.19	-0.64, 0.26	5.05
Cholesterol (mg)	57.67	-132.27, 247.62	4.04
Water (g)	6.00	-899.01, 911.02	4.55
Vitamin C (mg)	7.08	-96.75, 110.91	4.55
Calcium (mg)	-49.75	-444.09, 344.59	7.07
Copper (mg)	0.25	-0.44, 0.95	5.05
Iron (mg)	-0.99	-8.26, 6.28	4.55
Magnesium (mg)	-5.03	-122,11, 112.05	5.05
Manganese (mg)	-0.50	-2.57, 1.56	5.56
Phosphorus (mg)	-11.95	-426.76, 402.86	4.04
Potassium (mg)	171.35	-802.51, 1145.21	5.05
Sodium (mg)	639.14	-931.89, 2210.17	5.05
Zinc (mg)	0.37	-6.00, 6.73	3.03

Abbreviations: FFQ, food frequency questionnaire; LOA, limit of agreement.

* Percentage of subjects with values out of limits of agreement.
