# Supplementary figures and images for: Reproducibility and Relative Validity of a Short Food Frequency Questionnaire for Chinese Older Adults in Hong Kong
Source: Nutrients. 2024 Apr 11;16(8):1132. doi: 10.3390/nu16081132 (PMC11054710; doi:10.3390/nu16081132)

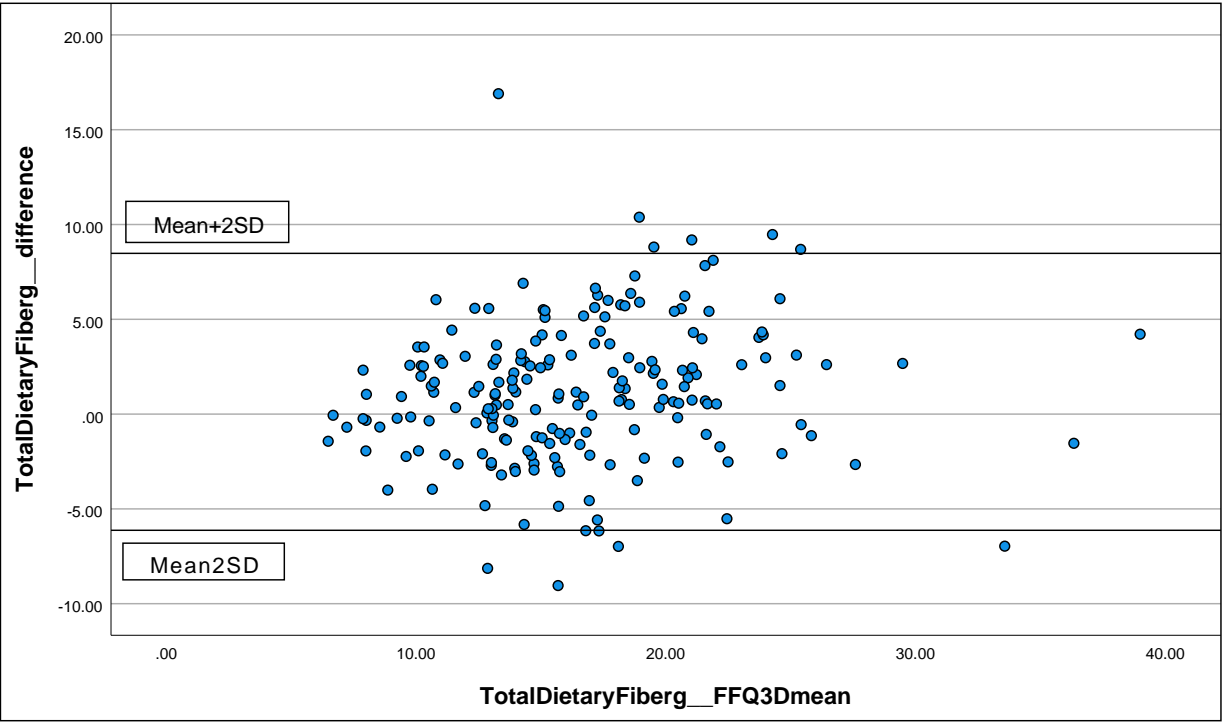

Supplement: Supplementary file 1 [file nutrients-16-01132-s001.zip › Figure S1. Total Dietary Fiber.pdf]

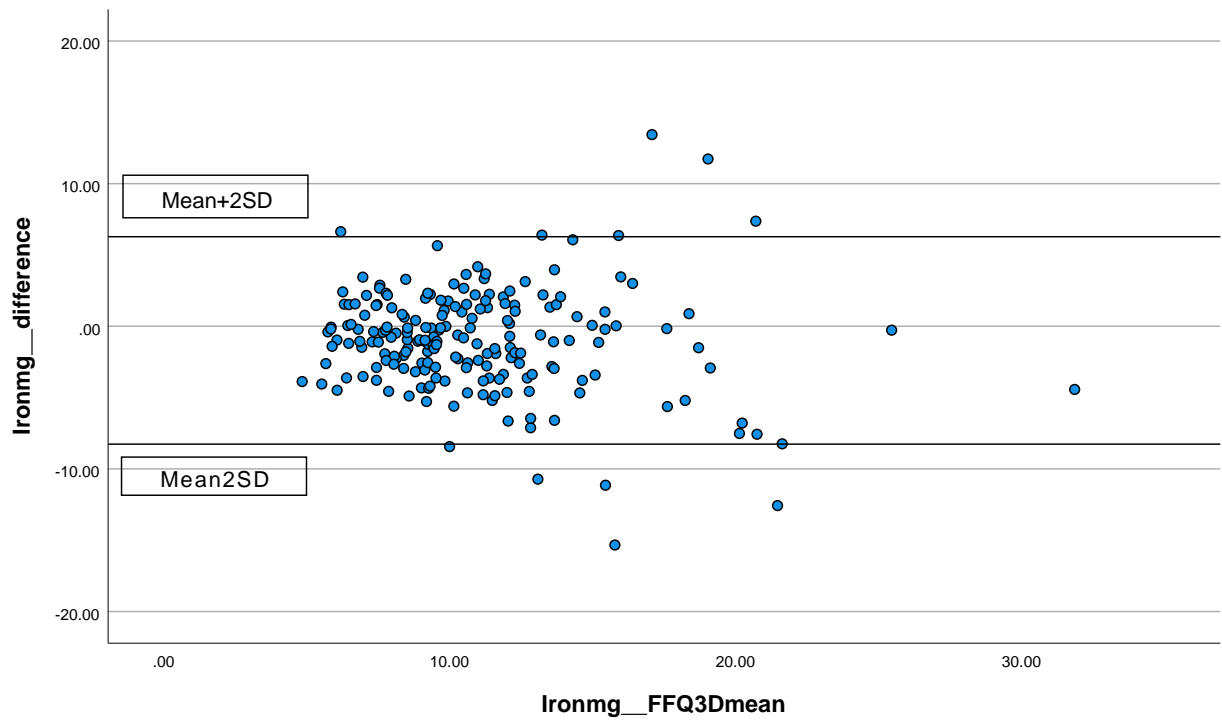

Supplement: Supplementary file 1 [file nutrients-16-01132-s001.zip › Figure S10. Iron.pdf]

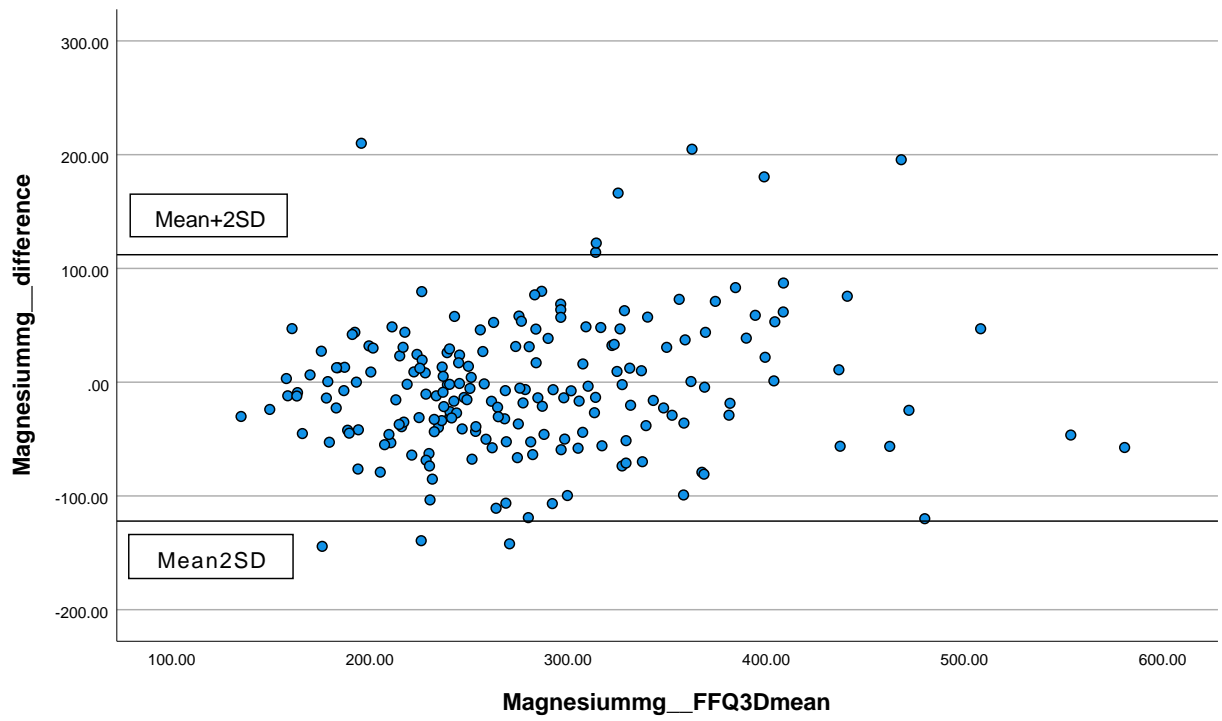

Supplement: Supplementary file 1 [file nutrients-16-01132-s001.zip › Figure S11. Magnesium.pdf]

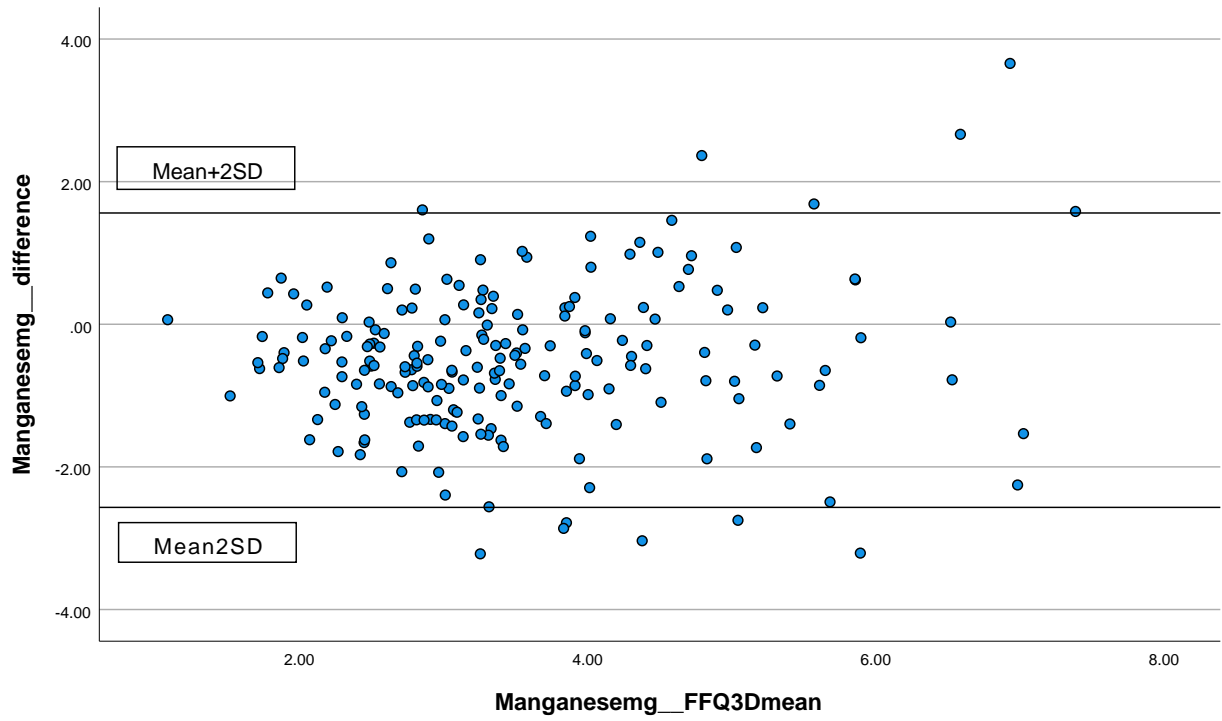

Supplement: Supplementary file 1 [file nutrients-16-01132-s001.zip › Figure S12. Manganese.pdf]

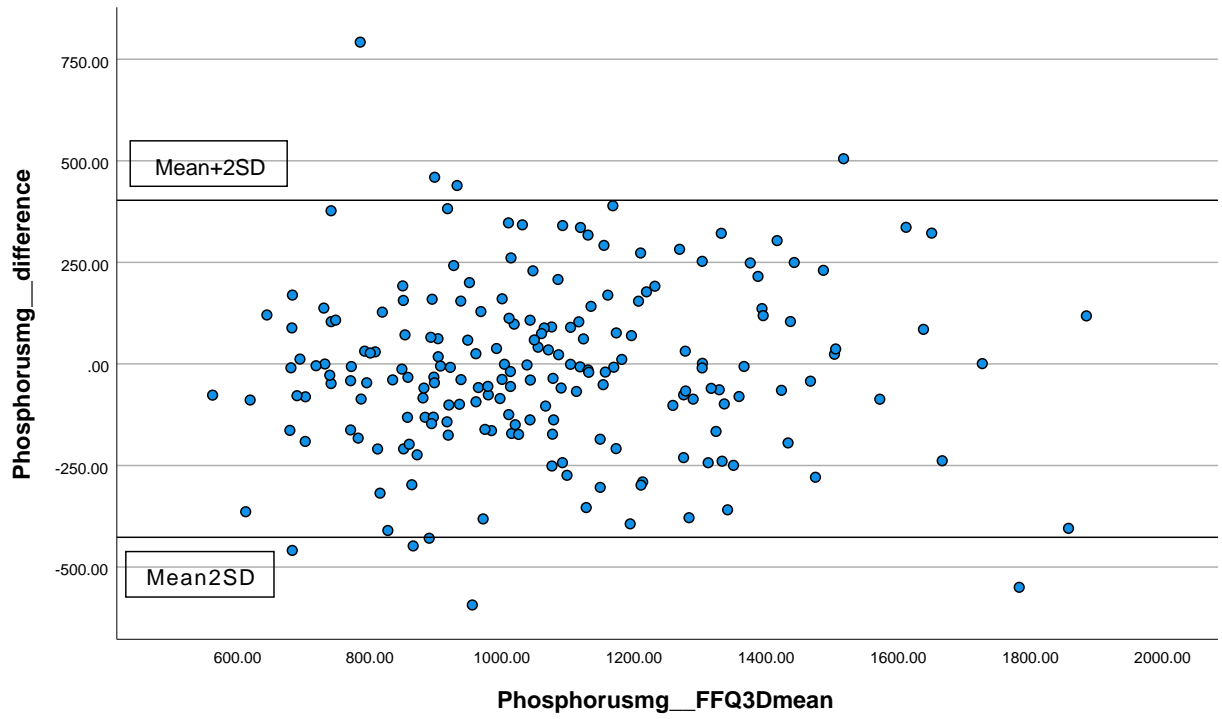

Supplement: Supplementary file 1 [file nutrients-16-01132-s001.zip › Figure S13. Phosphorus.pdf]

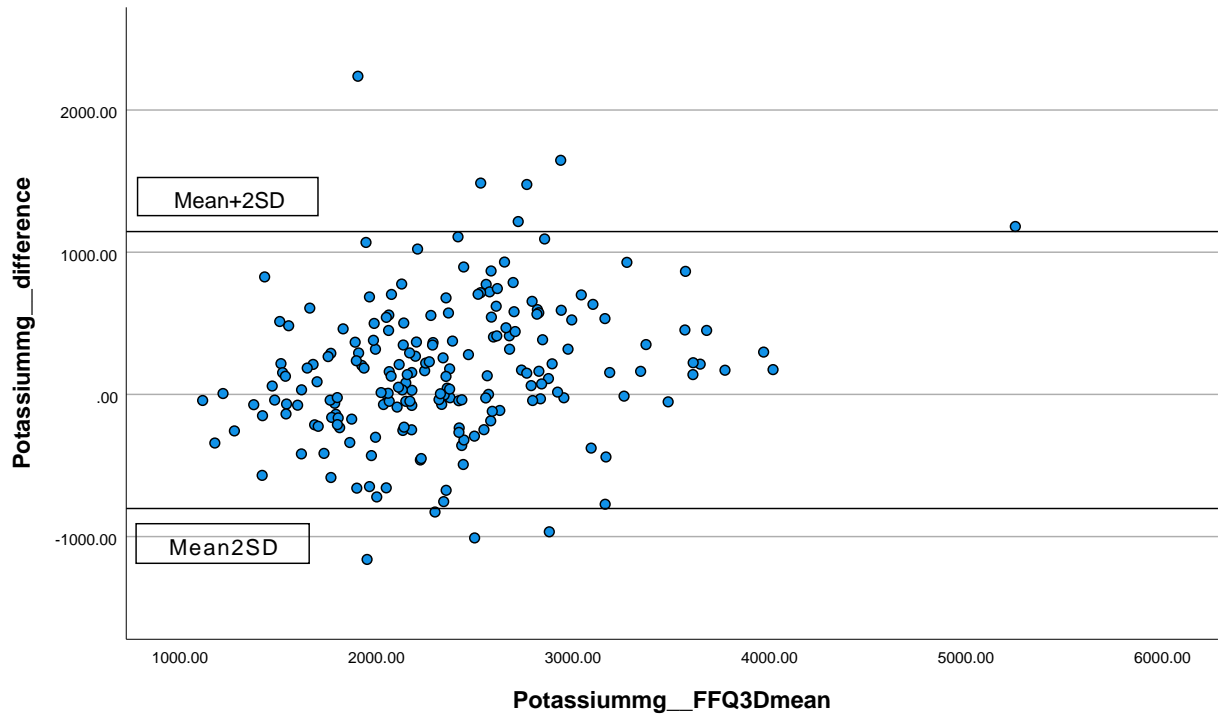

Supplement: Supplementary file 1 [file nutrients-16-01132-s001.zip › Figure S14. Potassium.pdf]

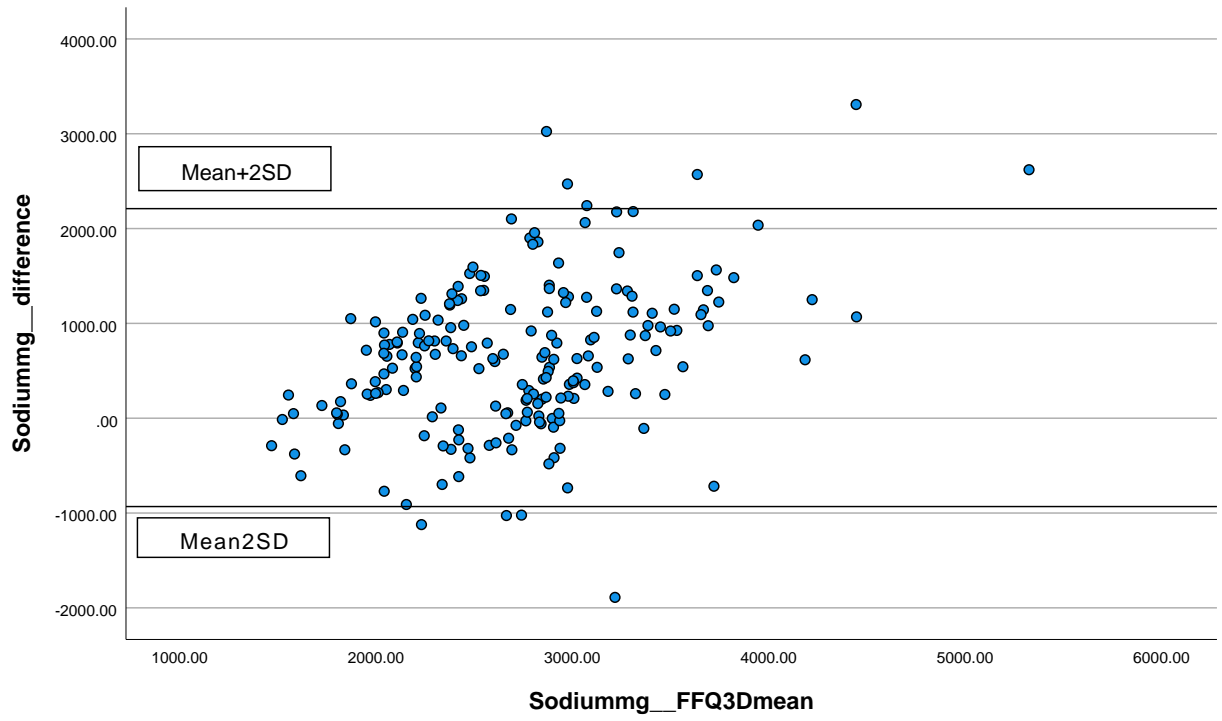

Supplement: Supplementary file 1 [file nutrients-16-01132-s001.zip › Figure S15. Sodium.pdf]

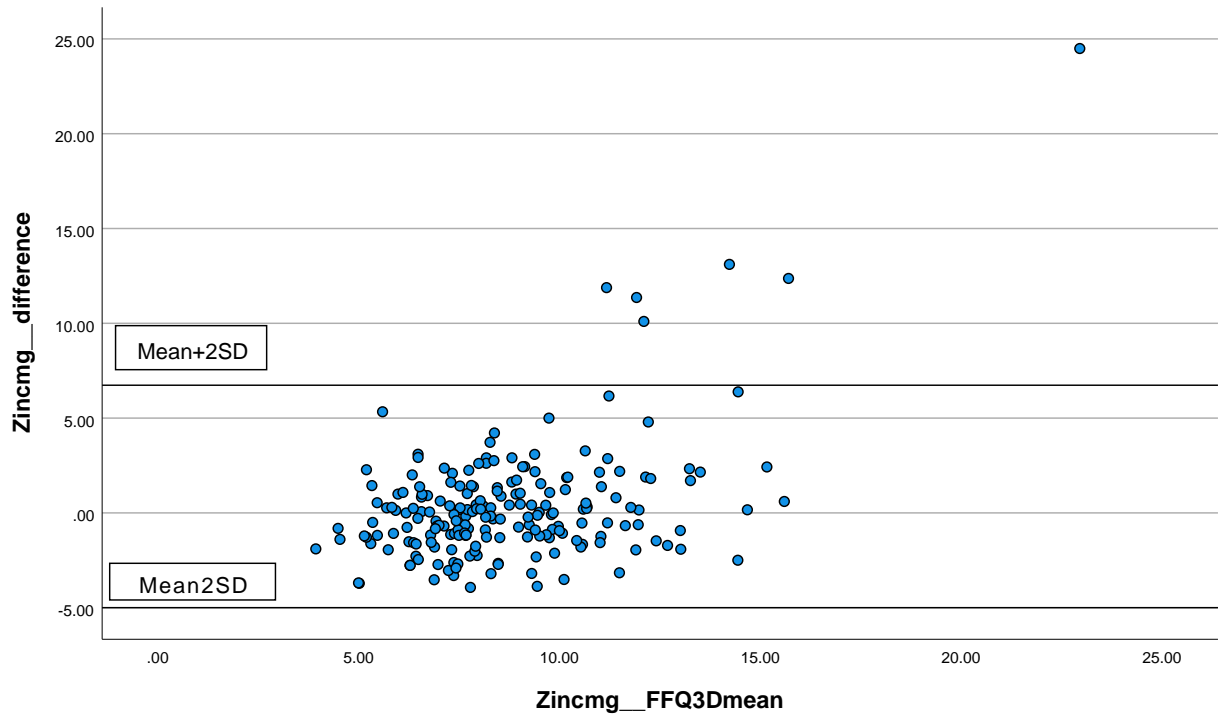

Supplement: Supplementary file 1 [file nutrients-16-01132-s001.zip › Figure S16. Zinc.pdf]

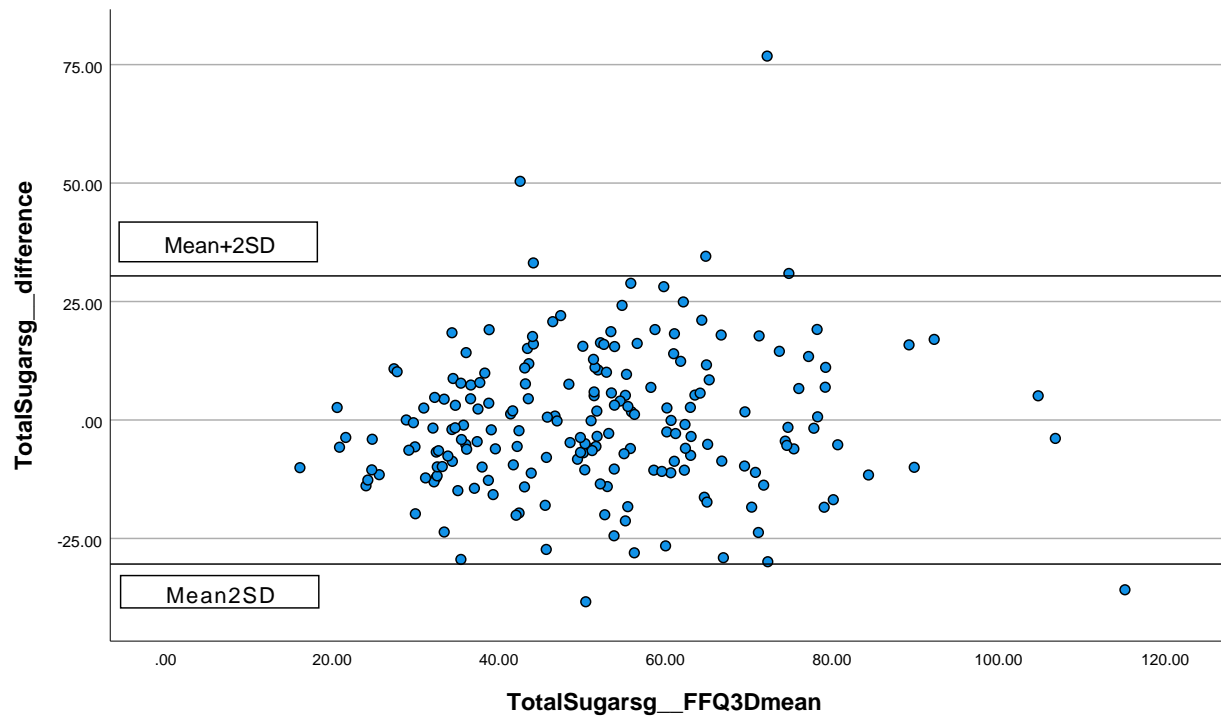

Supplement: Supplementary file 1 [file nutrients-16-01132-s001.zip › Figure S2. Total Sugars.pdf]

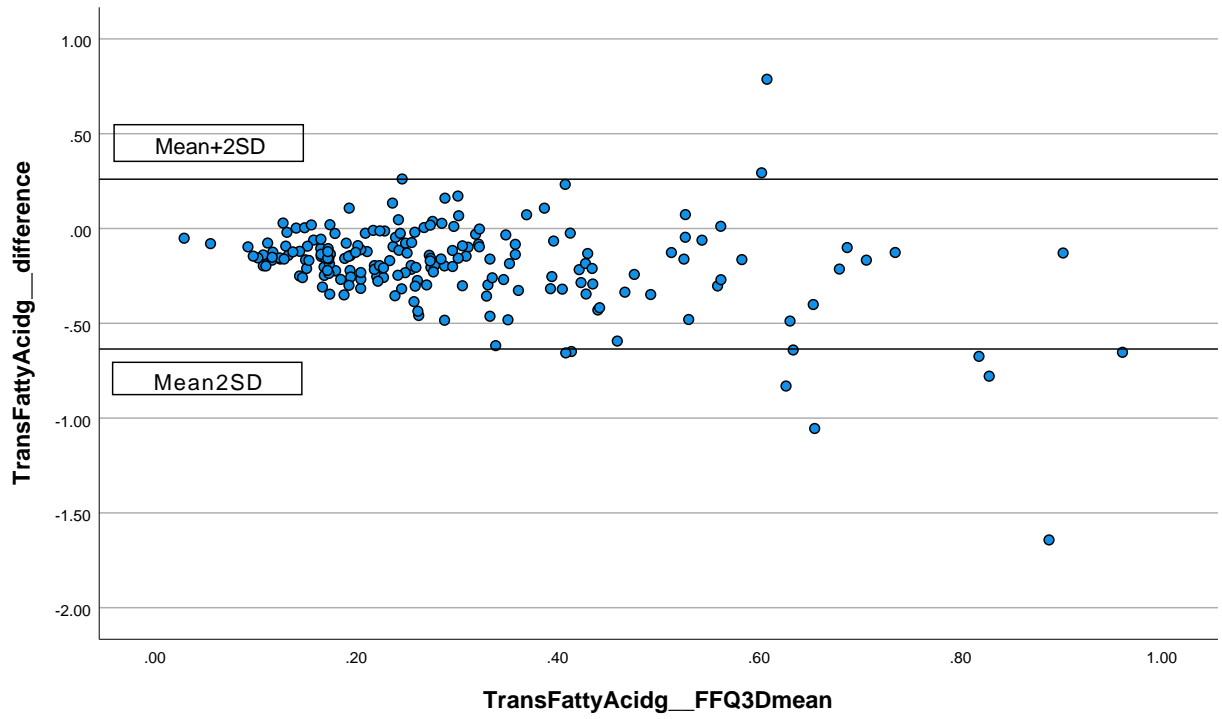

Supplement: Supplementary file 1 [file nutrients-16-01132-s001.zip › Figure S4. Trans-fat.pdf]

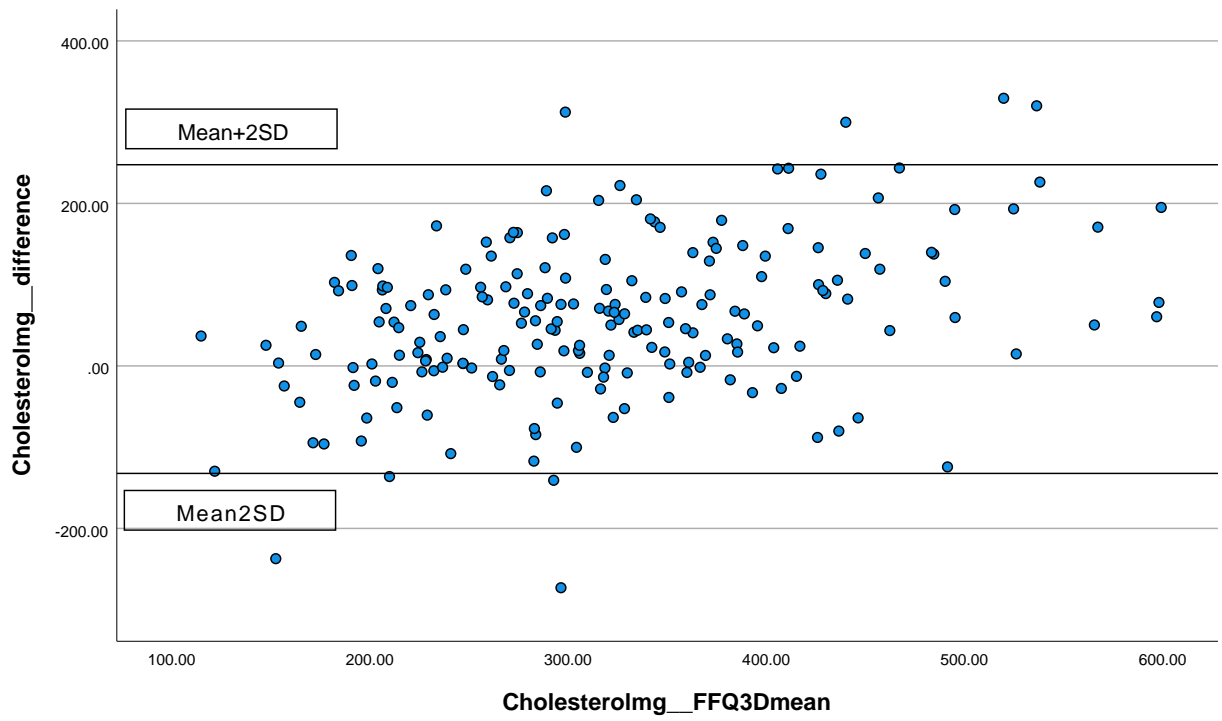

Supplement: Supplementary file 1 [file nutrients-16-01132-s001.zip › Figure S5. Cholesterol.pdf]

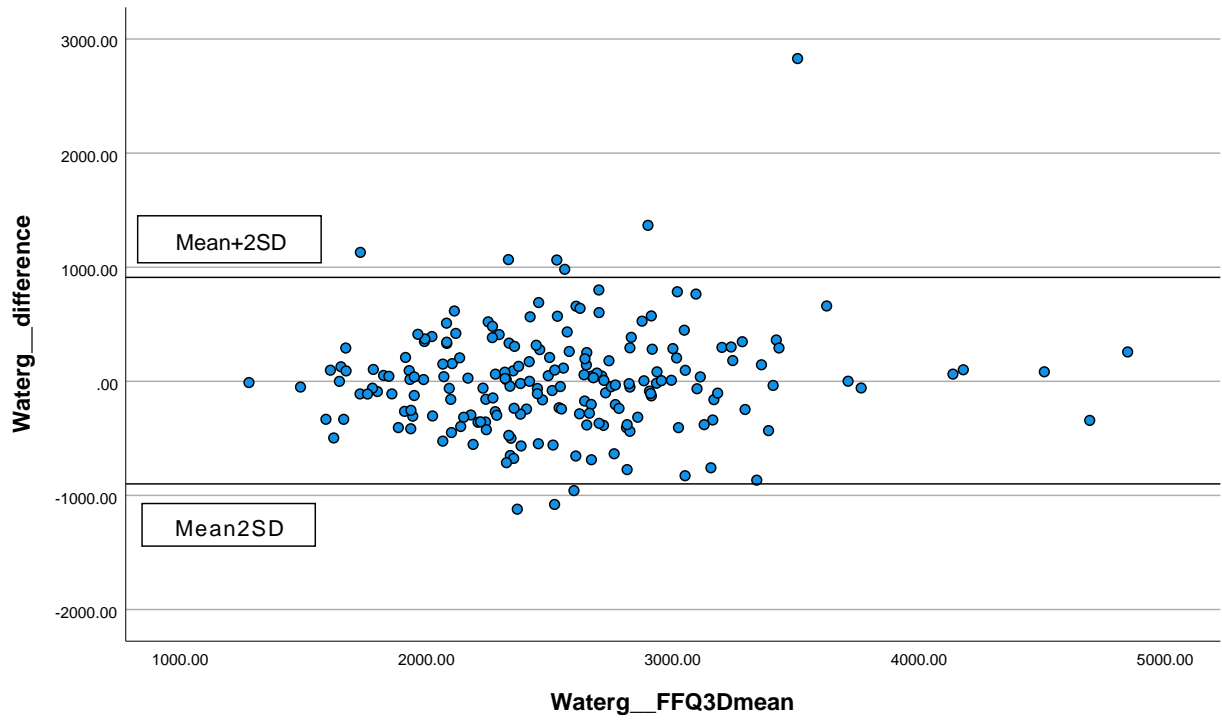

Supplement: Supplementary file 1 [file nutrients-16-01132-s001.zip › Figure S6. Water.pdf]

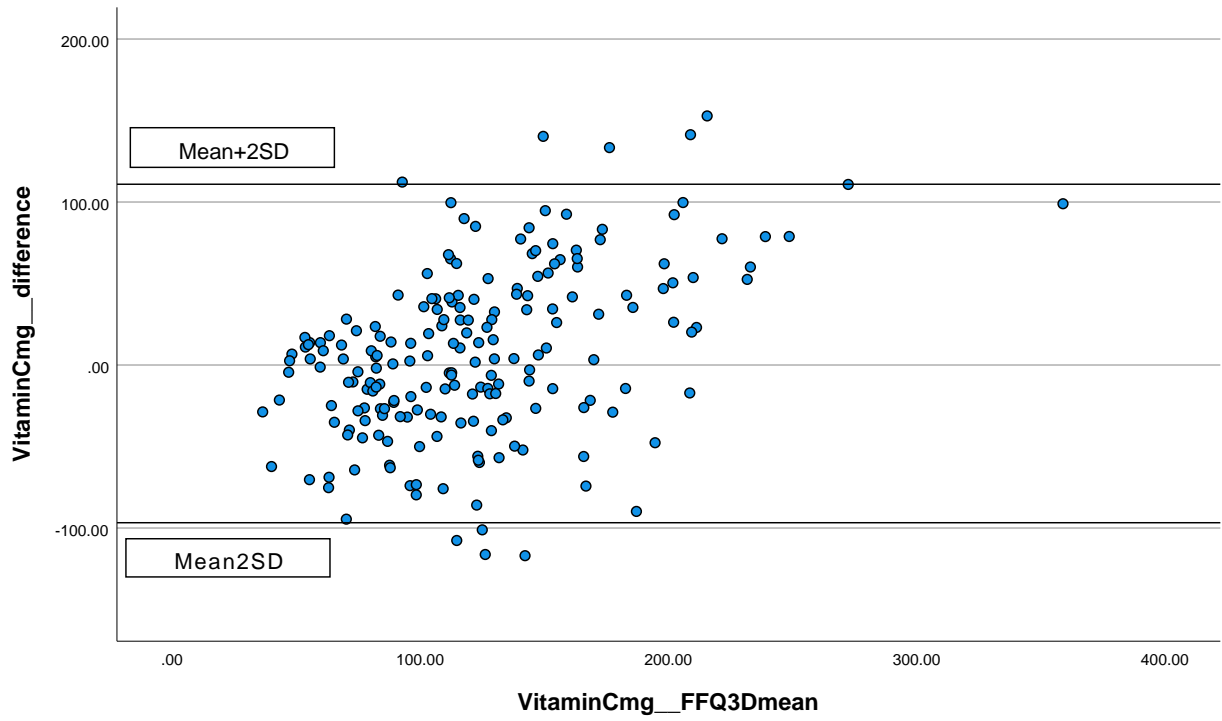

Supplement: Supplementary file 1 [file nutrients-16-01132-s001.zip › Figure S7. Vitamin C.pdf]

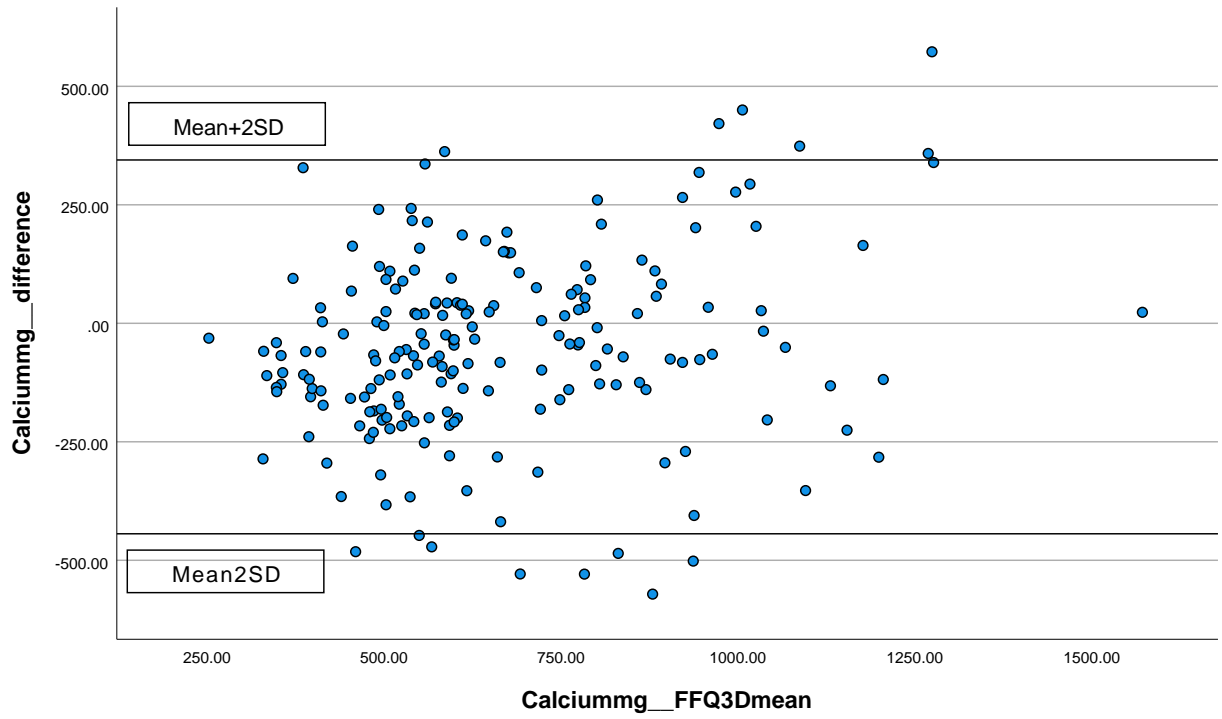

Supplement: Supplementary file 1 [file nutrients-16-01132-s001.zip › Figure S8. Calcium.pdf]

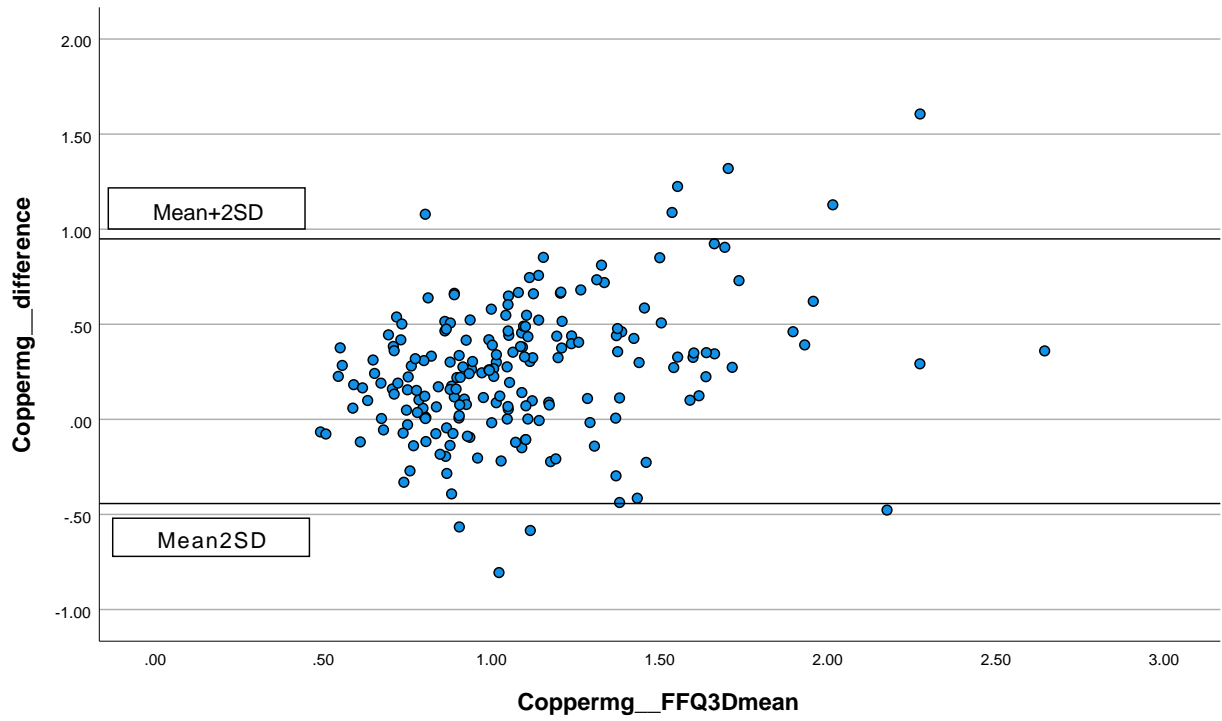

Supplement: Supplementary file 1 [file nutrients-16-01132-s001.zip › Figure S9. Copper.pdf]
