# Supplementary material for: Reproducibility and Relative Validity of a Short Food Frequency Questionnaire for Chinese Older Adults in Hong Kong
Source: Nutrients. 2024 Apr 11;16(8):1132. doi: 10.3390/nu16081132 (PMC11054710; doi:10.3390/nu16081132)
Supplement: Supplementary file 1 [file Figure S3. Saturated Fat.pdf › Figure S3. Saturated Fat.pdf]

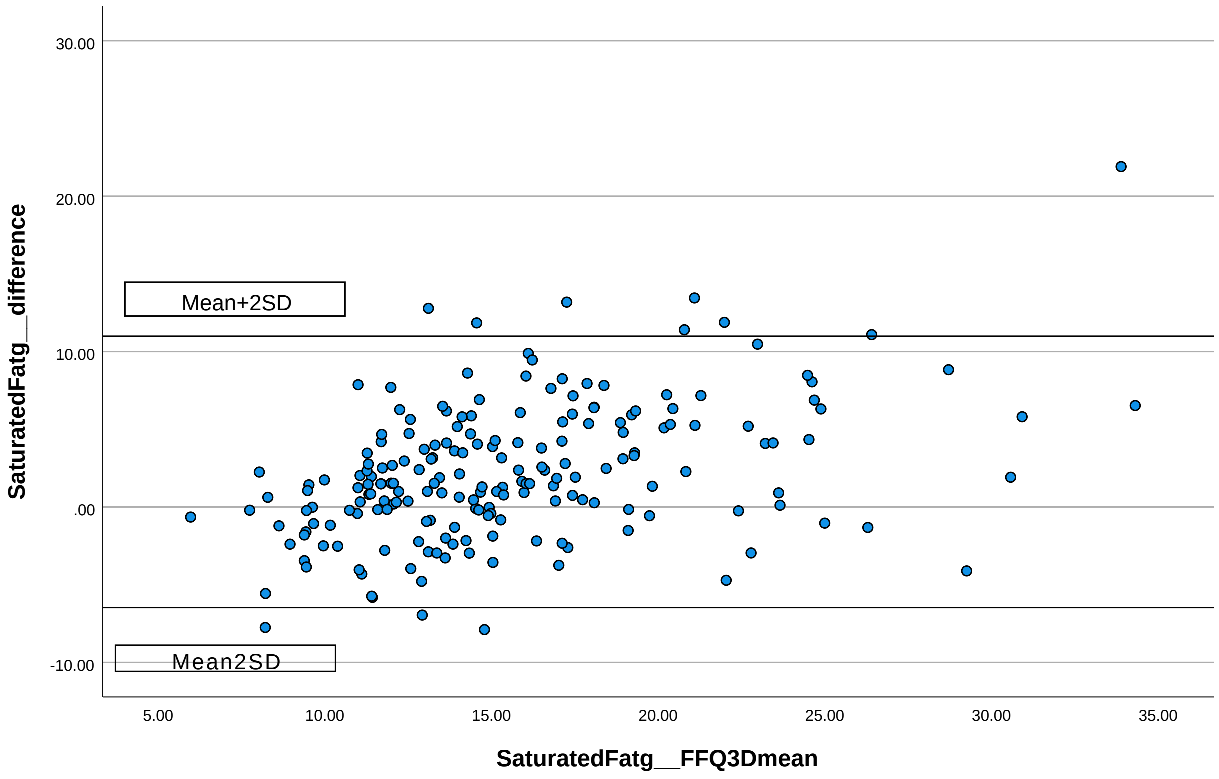
30.00
20.00
10.00
.00
-10.00
Mean+2SD
Mean2SD
SaturatedFatg__difference
5.00
10.00
15.00
20.00
25.00
30.00
35.00
SaturatedFatg__FFQ3Dmean
